# Exploring cellular memory molecules marking competent and active transcriptions

**DOI:** 10.1186/1471-2199-8-31

**Published:** 2007-05-10

**Authors:** Li Xin, Guo-Ling Zhou, Wei Song, Xue-Song Wu, Gong-Hong Wei, De-Long Hao, Xiang Lv, De-Pei Liu, Chih-Chuan Liang

**Affiliations:** 1 From National Laboratory of Medical Molecular Biology, Institute of Basic Medical Sciences, Chinese Academy of Medical Sciences and Peking Union Medical College, Beijing, 100005, PR China

## Abstract

**Background:**

Development in higher eukaryotes involves programmed gene expression. Cell type-specific gene expression is established during this process and is inherited in succeeding cell cycles. Higher eukaryotes have evolved elegant mechanisms by which committed gene-expression states are transmitted through numerous cell divisions. Previous studies have shown that both DNase I-sensitive sites and the basal transcription factor TFIID remain on silenced mitotic chromosomes, suggesting that certain trans-factors might act as bookmarks, maintaining the information and transmitting it to the next generation.

**Results:**

We used the mouse globin gene clusters as a model system to examine the retention of active information on M-phase chromosomes and its contribution to the persistence of transcriptional competence of these gene clusters in murine erythroleukemia cells. In cells arrested in mitosis, the erythroid-specific activator NF-E2p45 remained associated with its binding sites on the globin gene loci, while the other major erythroid factor, GATA-1, was removed from chromosome. Moreover, despite mitotic chromatin condensation, the distant regulatory regions and promoters of transcriptionally competent globin gene loci are marked by a preserved histone code consisting in active histone modifications such as H3 acetylation, H3-K4 dimethylation and K79 dimethylation. Further analysis showed that other active genes are also locally marked by the preserved active histone code throughout mitotic inactivation of transcription.

**Conclusion:**

Our results imply that certain kinds of specific protein factors and active histone modifications function as cellular memory markers for both competent and active genes during mitosis, and serve as a reactivated core for the resumption of transcription when the cells exit mitosis.

## Background

Higher eukaryotes contain several hundred cell types, each with a distinctive gene expression pattern. During differentiation, a committed expression pattern is set up through the sequential turning on and/or off of different sets of genes and this pattern is propagated through numerous symmetric cell divisions. Thus, multicellular organisms have evolved mechanisms for discriminating their gene expression states and maintaining these stages during cell differentiation. When eukaryotic cells enter mitosis, the chromatin becomes highly condensed, most transcription factors are displaced from the chromosomes and almost all transcription is abruptly turned off [1]. How do eukaryotic cells remember their previous transcriptional profiles during cell division, rather than having to re-establish the appropriate expression pattern from scratch at the onset of the next cell cycle? In most situations, all cells carry the same genetic material, the genomic DNA, throughout their lives, so chromatin-based mechanisms including interactions among DNA, histones and non-histone protein factors and modifications of DNA and chromatin proteins may account for the transmission of gene expression states through cell division.

Previous studies have shown that DNase I-sensitive sites mark active genes on silenced mitotic chromosomes [2,3]. This implies that certain factors withstand chromatin condensation and survive during mitosis to facilitate the rapid reactivation of genes in next cell cycle. A subsequent study has supported this 'bookmarking theory' [4]. The basal transcription factor TFIID is partially retained on gene promoters in mitotic chromosomes to serve as such 'bookmark', enabling active transcription to be resumed rapidly on exit from mitosis [5,6]. However, the promoter alone may not be enough for the required memory. Many distant regulatory elements have been shown to play important roles in maintaining proper tissue- and developmental stage-specific transcription [7,8]. Some of these elements are DNase I-sensitive before the gene is activated. For example, in multilineage progenitor cells, erythroid-specific DNase I-hypersensitive sites (HSs) have arisen in the distal regulatory elements of the mouse β-globin gene cluster [9]. An early study showed that these hypersensitive sites, once induced, can be propagated to daughter cells (over at least 20 generations) in the absence of the original inducer [10]. We therefore speculated that in addition to TFIID, certain tissue-specific transcription factors might serve as 'bookmarks' on distant regulatory regions, which also mediate the propagation of chromatin states during cell division. In MEL cells, transcription of globin genes is not activated before induction, yet these genes can retain their transcriptional competence for generations. In this study, we used the globin gene clusters as a model system to investigate whether specific transcription factors remain on the distal regulatory elements in M-phase chromosomes in transcriptionally competent MEL cells. We found that the p45 subunit of the erythroid-specific activator NF-E2 is partially retained on its specific binding sites during mitosis, but another activator, GATA-1, is not. This suggests that NF-E2 can function as a 'bookmark' to maintain the active competence of the local chromatin domain during cell division.

Recent advances in epigenetic studies have indicated that histone modification is also closely correlated with transcriptional regulation. Active histone modifications such as hyperacetylation and H3-K4 and H3-K79 methylation often mark transcriptionally active regions, whereas H3-K9 methylation is always related to transcriptional repression. Combinations of histone modifications could constitute a set of histone codes that are recognized by specific non-histone proteins to mediate specific chromatin functions [11,12]. Using two continuously active hepatic genes as models, a recent study revealed that localized histone hyperacetylation, H3-K4 tri- or di-methylation and H3-K79 dimethylation at the promoter and the 5' portion of the coding regions remain stable during mitotic chromatin inactivation [13]. This suggests that epigenetic factors such as histone modifications should also be considered potential memory markers. Here we have investigated the active histone modification status on both the α- and the β-globin gene promoters and the distant hypersensitive sites on mitotic chromosomes in MEL cells. The results show that multiple active histone modifications are stably preserved on the mitotic chromosomes.

## Results

### Erythroid-specific activator NF-E2, but not GATA-1, marking transcriptional competence of globin genes in MEL cell during mitosis

**Figure 1:**
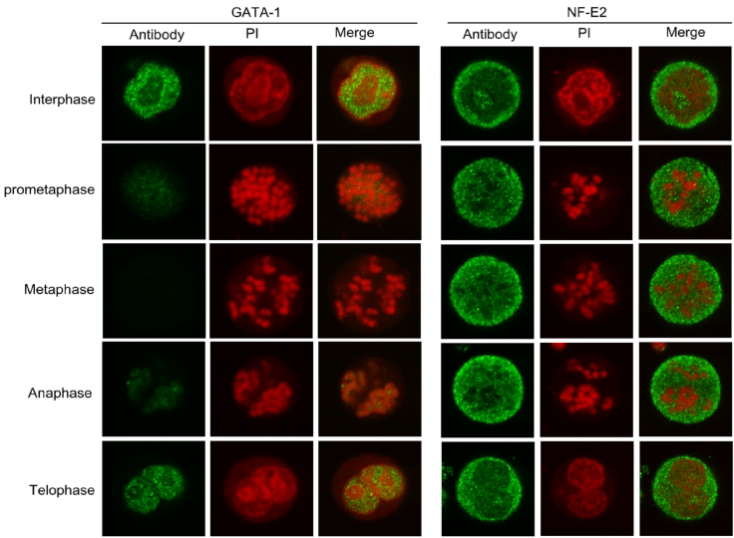
**Targeting of NF-E2p45 to chromatin compartment but displacing of GATA-1 from mitotic chromosomes in mitotic cells**. Cells were stained with antibodies against proteins indicated on the left followed by FITC-conjugated secondary antibodies (Left) and PI (Center). Right is the merge of both images. Examples of interphase cells and mitotic cells of different stages from MEL cells are shown.

In MEL cells, DNase I sensitivity and hypersensitivity have been established on globin gene loci [14], suggesting that they have obtained potential transcription activity. An early study also showed that hypersensitive sites, once been set up, can be propagated to daughter cells (over at least 20 generations) in the absence of the original inducer [10]. So we speculated that there might be some memory molecules, possibly involving protein factors or epigenetic marks, on regulatory sequences to help the propagation of transcription competence. There are some specific binding sites of erythroid-specific activators, such as GATA-1, NF-E2 and Fog-1, on globin gene clusters [15], whose occupancies largely contribute to the activation of globin genes. To explore the protein factors involved in the inheritance of transcriptional competence, we investigated the nuclear localization of GATA-1 and the activating subunit p45 of NF-E2 in mitotic and interphase cell by *in situ *immunofluorescence. It was found that GATA-1 mainly localized in interchromatin regions of cell nucleus and it can not be detected in nuclei of the mitotic cells by *in situ *immunofluorescence until parental nucleus divided into two progeny nuclei at telophase (Fig 1), indicating that GATA-1 was not retained on mitotic chromosome as a cellular memory mark during mitosis. In contrast, NF-E2p45, which is punctately organized in both cytoplasm and nucleus of the interphase cells, persists throughout mitosis (Fig 1), implying that it may be involved in the propagation of transcription competence of globin genes during cell division.

**Figure 2:**
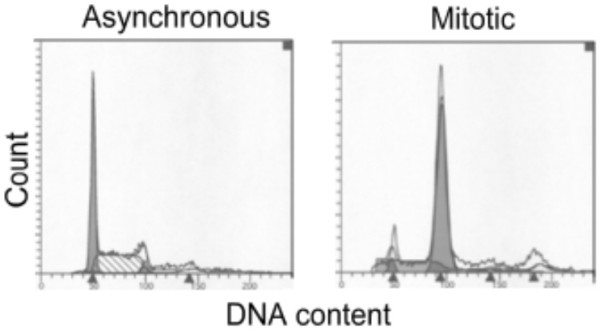
Flow cytometry analysis of asynchronous (A) and mitotic (M) MEL cells.

**Figure 3:**
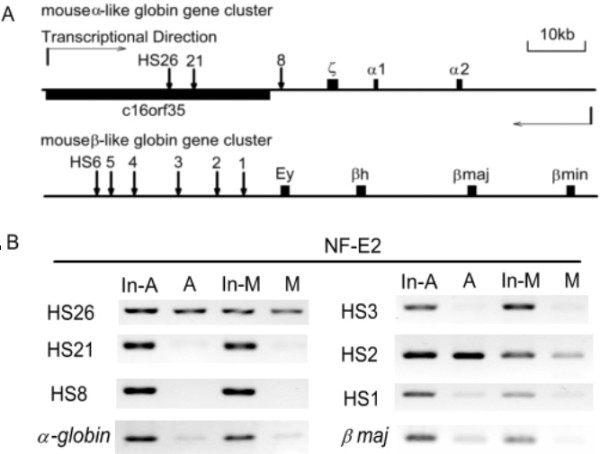
**The occupancies of NF-E2p45 on mouse α-like and β-like globin gene clusters in asynchronous (A) and mitotic (M) MEL cells**. A. The schematic presentation of mouse α-like and β-like globin gene clusters. Globin genes are shown in dark rectangle. The DNase I hypersensitive sites-HS26, HS21, HS8 of mouse α-globin locus and HS3, HS2, HS1 of mouse β-globin locus, which are analyzed in ChIP assay, are shown in arrow. B. The relative occupancies of NF-E2p45 at HS sites of globin clusters in A and M cell populations were analyzed after normalizing the DNA amount differences in A and M cell populations through DNA input (In-A and In-M).

In order to confirm this possibility, we arrested murine erythroleukemia (MEL) cells into mitosis by treating the cells with nocodazole [3]. The mitotic index of cell population after treatment was confirmed to be 92–96% by flow cytometry analysis (Fig 2). The results also showed that a proportion of ~5% mitotic cells, about 50% G0/G1-phase and 45% S-phase cells, respectively, is present in asynchronous MEL cell population (Fig 2). The two cell populations were analysed by ChIP to compare the occupancies of NF-E2p45 on distant hypersensitive sites of two mouse globin gene clusters (Fig 3A). The result showed that NF-E2p45 could be specifically recruited to its binding sites HS26 and HS2 in interphase cells (Fig 3B). In mitotic cells it is still tightly associated with HS26 and HS2 (Fig 3B), indicating that NFE2p45 can be retained on mitotic chromosomes to maintain the hypersensitivity of the localized regulatory regions of two mouse globin gene clusters. Its retention, moreover, marks the transcriptional competence of globin genes during MEL cell division.

### Epigenetic memory marking transcriptionally competent globin genes

**Figure 4:**
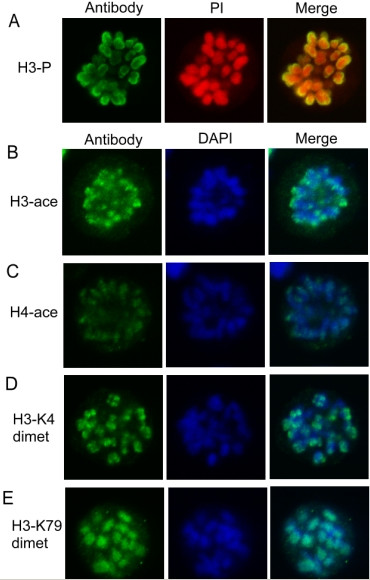
**Flow cytometry analysis of asynchronous (A) and mitotic (M) MEL cells**. Cells were stained with antibodies against histone modifications indicated on the left followed by FITC-conjugated secondary antibodies (Left) and PI (red) or DAPI (blue) (Center). Right is the merge of both images. Mitosis marker-H3 phosphorylation (A) and active histone modifications-H3 acetylation (B) and H4 acetylation (C), H3 K4 dimethylation (D) and K79 dimethylation (E) were observed on mitotic chromosomes by in situ immunofluorenscence.

**Figure 5:**
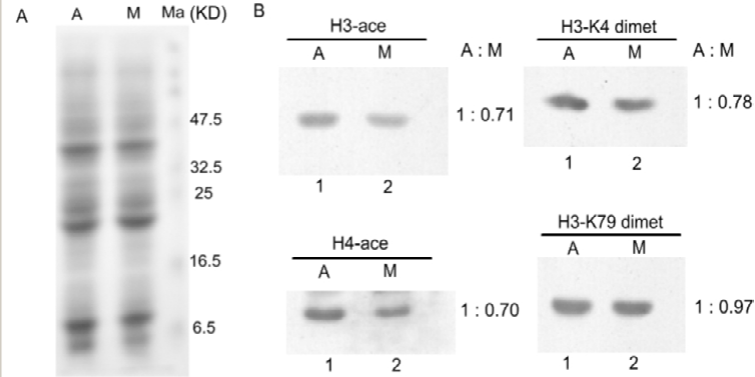
**The comparative analysis of global histone modificartions in asynchronous (A) and mitotic (M) MEL cells through western blotting**. A. Histones prepared from A and M cell extracts were electrophoretically separated by SDS-polyacrylamide gel electrophoresis and stained with Coomassie Blue. B. The whole level of H3 and H4 acetylation, H3-K4 dimethylation in M cells is decreased compared to that in A cells, but H3-K79 dimethylation is stable.

**Figure 6:**
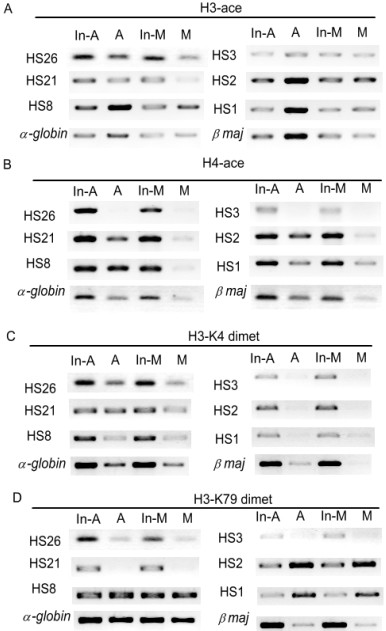
**The distribution of active histone modifications on mouse α-like and β-like globin gene clusters in asynchronized (A) and mitotic (M) MEL cells**. A, B. The changes of H3 and H4 acetylation level on the distant regulatory elements of globin loci in A and M cell populations. C, D. The changes of H3-K4 and H3-K79 dimethylataion on the distant regulatory elements of globin loci in A and M cell populations. The relative level of histone modifications at HS sites of globin clusters in A and M cell populations were analyzed after normalizing the DNA amount differences in A and M cell populations through DNA input (In-A and In-M).

Besides protein factors, histone modification is recently identified as an efficient epigenetic factor involved in gene expression regulation through altering chromatin structure. Do the eukaryotic cells preserve some epigenetic marks on mitotic chromosomes to maintain its gene expression states? To verify the above-proposed possibility, we first observed the nuclear localization of four kinds of active histone modification including H3 acetylation, H4 acetylation, H3-K4 dimethylation and H3-K79 dimethylation in mitotic cells by *in situ *immunofluorescence. The results showed that all of the four modifications are retained on the mitotic chromosomes to some extent (Fig 4). We further detected the global changes of those modifications in both asynchronized and mitotic arrested MEL cell populations by western blotting. After correcting the variation in gel-loading and normalizing for the background, we found that there is a partial loss (about 20–30%) of H3 acetylation, H4 acetylation and H3-K4 dimethylation in mitotic cell extraction compared to those in asynchronous cell extraction but no obvious loss of H3-K79 dimethylation (Fig 5). This indicates that only part of histone acetylation and H3-K4 dimethylation signals can be inherited to mitotic chromatin through DNA replication-coupled chromatin assembly process. What is the detailed distribution of these preserved modifications on the mitotic chromosome? We therefore analysed the histone modification status across the distant hypersensitive sites and the adjacent promoter regions of mouse α- and β-globin genes (Fig 3A) during mitosis by the comparative ChIP analysis of the above mentioned asynchronous and mitotic arrested MEL cell populations. The results showed that HS26, HS21 and HS8 of mouse α-globin locus and HS3, HS2 and HS1 of mouse β-globin locus, as well as the promoters of α-globin and *βmaj *are acetylated at H3 and all of them except HS26 and HS3 are acetylated at H4 in asynchronous cells (Fig 6A, 6B). Higher H3-K4 dimethylation at HSs and α-globin promoter was noticed when compared to that at β-globin promoter(Fig 6C). HS8 and α-globin promoter are hypermethylated at H3-K79, but HS26 and HS21 show trace of signal. HS2 and HS1 of LCR are hypermethylated at H3-K79, while HS3 and βmaj promoter are just slightly methylated at H3-K79 (Fig 6D). In mitotic cells, the levels of H3 and H4 acetylation, H3-K4 dimethylation dropped at many analyzed regions, while H3-K79 dimethylation at all the analysed regions remain stable compared to those in asynchronous cells. Moreover H3-K79 dimethylation level on mitotic chromosomes is comparable to that in asynchronous cells, which consist with their chromatin states (Fig 6). The results suggested that, despite of some losses, the established active histone code at distant regulatory regions and globin gene promoters before mitotic chromatin inactivation can be stably inherited to mitotic chromosomes to mark the transcriptional competence of mouse globin genes in MEL cells.

### Epigenetic memory marking the active transcription states of the genes

**Figure 7:**
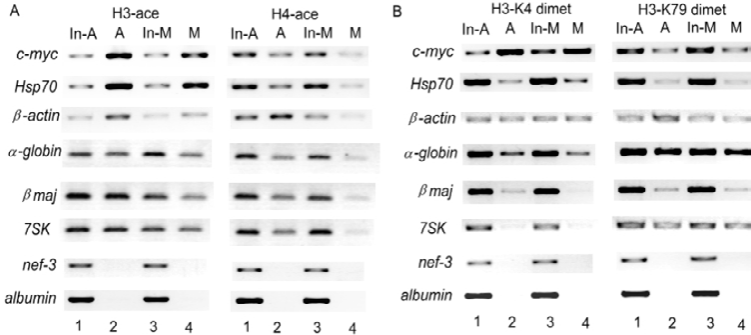
**The distribution of active histone modifications on differently active gene promoters in asynchronized (A) and mitotic (M) MEL cells**. A. H3 and H4 acetylation. B. H3-K4 and H3-K79 dimethylation Comparative. ChIP analyses were performed with the equivalent DNA input amounts of chromatin from asynchronous and mitotic cells, then analyzed by PCR (In-A and In-M). PCR primer pairs flank the promoter regions of transcriptionally active, competent and inactive genes. The relative level of histone modifications at promoters of the individual genes in A and M cell populations were analyzed after normalizing the DNA amount differences in A and M cell populations through DNA input (In-A and In-M).

Can these epigenetic marks note the different transcription states of thegenes during mitosis? We next performed the comparative chromatin immunoprecipitation (ChIP) analysis for the promoter regions of the genes with different transcription states. These genes include (1) *c-myc*, *hsp70 *and *β-actin*, which are universally transcribed by RNA polymerase II (RNAP II); (2) an actively RNAP III-transcribed gene (*7SK*), and (4) *nef-3 *and *albumin*, which are tissue-specifically silenced in MEL cells. The results showed that promoter region of the actively transcribed genes like *c-myc*, *hsp70*, *β-actin *and *7SK *are hyperacetylated, while inactive genes, *nef-3 *and *albumin*, are hypoacetylated at both H3 and H4 in asynchronous MEL cells (Fig 7A). In synchronized mitotic cells, transcriptionally active genes keep obviously higher levels of histone acetylation at both H3 and H4 than those of inactive genes,(Fig 7A). The fact that, during mitotic chromatin inactivation, the levels of remnant histone acetylation at the promoter regions of the analysed genes depend on their previous expression states strongly suggests that the localized histone acetylation at transcriptionally competent or active gene regions can be taken as the useful epigenetic memory marks to imprint the previous expression states during cell division.

To investigate whether active histone modifications-H3-K4 dimethylation and K79 dimethylation are preserved at active regions during mitosis, we performed the similarly comparative ChIP analysis. The results showed that these two kinds of modification are also mainly confined to active gene promoters (Fig 7B). In asynchronous cells, the fully activated genes-*c-myc *and *β-actin *are hyperdimethylated at H3-K4; incompletely activated gene *hsp70 *are moderately dimethylated at H3-K4. Although RNA polymerase III-transcribed gene *7SK *is active, similar to inactive genes as *nef-3 *and *albumin*, there is tiny enrichment of H3-K4 dimethylation on the gene. However, H3-K79 dimethylation, despite the moderate level, is distributed on the promoters of all the analyzed active genes regardless of the RNA polymerase used for transcription. In mitotic cells, these two kinds of active histone modification, similar to histone acetylation, are also preserved on the previously active gene promoter regions and the preserved levels are corresponding with gene expression states. Nevertheless, there is some decrease for H3-K4 dimethylation but no obvious change for H3-K79 dimethylation (Fig 7B). These data indicate that the localized H3-K4 dimethylation and K79 dimethylation at transcriptionally active regions can also serve as molecular memory marks along with histone acetylation. The above results also confirmed that the epigenetic marks composed of active histone modification combinations and localized at active gene regions can be transmitted to mitotic chromosomes for the maintenance of active gene expression states.

## Discussion

Studies on the α- and β-globin genes suggest that certain mechanisms in higher eukaryotic cells fix the stochastic expression patterns during cellular differentiation. Although there is a tight balance in the expression of α- and β-globin genes, these expression patterns are consistent with the predictions of the stochastic model [26,27]. Analysis has revealed an imbalance between 2α- and 2β-globin gene expression in both cytoplasm and nucleus in a significant proportion of erythroid cells. Further elegant experiments have demonstrated that these stochastic expression patterns are established prior to transcriptional activation. Importantly, both active and silenced expression patterns are clonally inherited [28]. The inherited stochastic patterns cannot be explained simply by random molecular encounters or fluctuations in the transitions between conformational states of a macromolecule, or by the amplification of feedback loops in a transcription factor network. Differentiated cells appear to have developed important mechanisms for remembering both transcriptionally active and silent states.

Previous studies have indicated that when cells enter mitosis, almost all transcription factors are peeled off the chromatin because the transcription complexes are disrupted [1]. However, it has been found that TFIID is retained at active gene promoters during mitotic chromatin inactivation [6]. During the process of transcription complex assembly, TFIID interacts first with the core promoter, so its retention on the mitotic chromosomes is an economical way of resuming transcription after mitosis. It has been observed that two enzyme classes, the histone acetyltransferases (HATs) and the histone deacetylases (HDACs), are spatially reorganized and displaced from the condensing chromosomes as the cells progress through mitosis [16]. In another study [13] it was shown that histone acetylase (CBP, PCAF), chromatin remodeling complex component (Brg-1), SNF2H and FACT, along with RNA pol-II at the HNF-4, HNF-1 and albumin genes, are all dissociated from the chromatin during mitosis but re-recruited on mitotic exit [13], suggesting that they have roles in the reactivation of transcription.

By employing globin genes as a model system, we found in this study that the tissue-specific factor NF-E2p45, which is crucial for activating globin genes, is preserved on mitotic chromosomes but GATA-1 is not. This suggests that during cell division, NF-E2p45 can serve as a molecular memory marker that maintains the locally hypersensitive state of the globin gene clusters. Previous studies have shown that GATA-1 is involved in regulating transcription by undergoing modifications such as acetylation, phosphorylation and sumoylation, without the recruitment of other partners [17-19]. Chromatin remodeling complexes and histone modification enzymes, which are displaced from mitotic chromosomes, depend on other protein factors to reunite them with the chromatin [13]. During globin gene activation, NF-E2 plays a key role in recruiting other protein factors such as TAF_II_130 and CBP, a coactivator of histone acetyltransferase activity [20]. TFIID retained on the mitotic chromosomes could also recruit other basal transcription factors when transcription is activated. Hence, we predict that higher eukaryotes have evolved mechanisms for preserving certain proteins on mitotic chromosomes, which may have common functional features for recruiting other protein factors to ensure that transcription is efficiently reactivated at the onset of each cell cycle. It will therefore be worth exploring other similar protein factors and their memory functions in order to elucidate cellular memory mechanisms.

The binding to DNA of many protein factors involved in the regulation of gene expression often depends on alteration of the local chromatin structure, mediated by post-translational modifications of the terminal residues of histones. As a result, different histone modifications segregate the chromatin into different territories. Active chromatin regions are often hyperacetylated at histones H3 and H4 and hypermethylated at H3-K4 and H3-K79 [21]. However, active histone modifications are highly dynamic. When chromatin condenses during mitosis, therefore, do these modifications function as memory 'determinants' to mark different states of gene expression? In this study, we selected transcriptionally competent globin gene clusters and genes in different transcriptional states to investigate the potential role of four active histone modifications (H3 and H4 acetylation, H3-K4 dimethylation and K79 dimethylation) as cellular memory markers during mitosis, using comparative ChIP analysis of both asynchronous and mitotic MEL cell populations. In asynchronous cells, multiple combinations of the four histone modifications are located at the distant HSs of the globin gene clusters and the gene promoter regions, and they mark different chromatin states of the genes. For example, fully activated *c-myc *and *β-actin *are hyperacetylated at both H3 and H4 and hyperdimethylated at H3-K4; incompletely activated *hsp70 *is hyperacetylated at both H3 and H4 and moderately dimethylated at H3-K4; potentially transcriptional α-globin and *βmaj *are moderately acetylated and dimethylated at H3-K4; the RNA polymerase III-transcribed gene *7SK *is hyperacetylated at both H3 and H4 but not dimethylated at H3-K4. Both transcriptionally active and competent genes, including RNA polymerase II- and III-transcribed genes, are all moderately methylated at H3-K79. However, inactive genes have less of these active modifications. In mitotic cells, some active modifications, especially H3 acetylation and H3-K4 dimethylation, are well preserved on the mitotic chromosomes. These preserved active histone modifications provide epigenetic markers by which gene expression states and local chromatin states can be inherited through mitosis. For the distant regulatory elements, which play important roles in switching on many tissue- or developmental stage-specific genes and maintaining their normal transcription during development and differentiation, localized active histone modifications, together with certain transcription factors, also provide important memory markers for maintaining local chromatin states during mitosis and hence facilitating and stabilizing differentiation and development. Moreover, the preserved epigenetic markers might contribute to create a unique chromatin conformation and provide a re-activating core for the resumption of transcription in the next cell cycle. They may also have roles in distinguishing transcriptionally active from repressed regions, even in the absence of trans-acting factors, at the onset of the next cell cycle.

Moreover, active modifications such as H3-K4 methylation and H3 acetylation are enriched in the H3.3 variant of *Drosophila*. In contrast, H3-K9 dimethylation, the marker for repressed genes, is enriched in H3 [22,23]. Another recent study showed that H3.3 is enriched in the promoters of active genes [24]. Using the mouse λ5-VpreB1 locus as a model system, immuno-FISH analysis of mitotic cells revealed that H3.3 is strongly marked at the active λ5 gene on metaphase chromosomes. Moreover, this region is hyperacetylated at H3-K9 and -K14 and di- and tri-methylated at H3-K4 [25]. These results strongly suggest that during mitosis, H3.3 might function as another epigenetic memory marker for maintaining transcriptionally active states along with active histone modification. Nevertheless, our speculation needs to be verified by testing more gene loci. It was also found that H3.3 incorporation into chromatin and its removal from chromatin depend on transcription [24]. Therefore, it will be worth exploring how H3.3 coordinates with multiple active histone modifications to mediate transcriptional memory.

## Conclusion

We conclude that both specific transacting factors and active histone modifications could function as cellular memory markers for competent and active genes during mitosis, and could serve as a reactivated core for the resumption of transcription after the cells exit mitosis.

## Methods

### Cell culture and mitotic arrest

Murine erythroleukemia (MEL) cells were grown in Dulbecco's modified Eagle's medium (DMEM), supplemented with 10% fetal calf serum. The cells were arrested at the G2/M phase by treating with nocodazole (which synchronizes cells to metaphase [3] (0.1 μg ml^-1^, Sigma) for 16 h. The mitotic index was determined by flow cytometry.

### Immunofluorescence

The synchronized MEL cells were laid on the cover slips, and then fixed with 3.0% paraformaldehyde at room temperature for 15 min. Subsequently, cells were washed three times in PBS and then permeabilized in PBS containing 0.1% Triton X-100 for 5 min. After that, cells were blocked with 3%BSA/PBS containing 2% goat serum followed by primary antibody incubation at 22°C for 2 h including the antibodies against GATA-1 (N6) and NF-E2p45 (C-19) (Santa Cruz Biotechnology), histone H3 phosphorylation (06–570), H3 acetylation (06–599), H4 acetylation (06–866), H3-K4 dimethylation (07–030), H3-K79 dimethylation (07–366) (Upstate), which are diluted in blocking solution. The cover slips were washed three times in PBS, and then incubated in the presence of FITC-conjugated-anti-rabbit immunoglobin G (IgG) (F0382; Sigma) for a further 60 min. Cover slips were washed again in PBS, incubated in 1 mg/ml DAPI or PI for 1 min and then mounted on slides with a 90% glycerol-PBS (pH 7.5)-based medium containing 1 mg/ml paraphenylenediamine and sealed with nail polish. Immunostaining signal of cell preparations was recorded by Laser Screen Confocal Microscope (Bio-Rad, Randiance 2100) attached to a laser sharp software.

### Acid extraction of total histones and Western Blotting assay

The asynchronous MEL and mitosis-arrested MEL cell populations were concentrated by centrifugation (800 × g) and washed twice with 1 × PBS. The washed and concentrated cells were resuspended in 5 volumes of ice-cold buffer (10 mM HEPES, pH 7.9, 1.5 mM MgCl_2_, 10 mM KCl, 0.5 mM dithiothreitol, 1.5 mM phenylmethylsulfonyl fluoride; Both dithiothreitol and phenylmethylsulfonyl fluoride were added just prior to the use of buffer). Concentrated sulfuric acid was added to the suspension to give a concentration of 0.4 N. After incubation on ice for 30 min, the suspension was centrifuged at 11,000 × g at 4°C for 15 min. The resultant supernatant was decanted into another tube and mixed with 1 ml of acetone. After overnight incubation at -20°C, the precipitated proteins were collected by centrifugation at 11,000 × g at 4°C for 15 min. The pellet was washed twice with 1 ml of acetone and air-dried. The dried pellet was then mixed with 2× SDS-polyacrylamide gel electrophoresis (SDS-PAGE) loading buffer. After incubation at 4°C overnight, the supernatant was decanted into another tube and quantified for the western blotting assay.

For western blotting, the samples were boiled for 5 min and loaded onto a 15% SDS-polyacrylamide gel. The electrophoretically separated proteins were either stained with Coomassie Brilliant Blue or transferred to immnobilon-P membrane (Millipore). The membrane filters were blocked with PBST (1× PBS, 0.05% Tween 20) containing 5% nonfat milk powder (PBST-Milk) at room temperature for 1 h and then washed three times each with PBS-T for 15 min. The primary antibody (anti-H3 acetylation: 1:10000, anti-H4 acetylation: 1:500, anti-H3-K4 dimethylation: 1:1000, H3-K79 dimethylation: 1:1000) diluted in PBST-Milk was applied at room temperature for 2 h or with agitation at 4°C overnight. After three washes with PBS-T, the secondary antibody-goat anti-rabbit HRP conjugated IgG(12–348, Upstate)(1:5000 dilution) in PBST-Milk was applied at room temperature with agitation for 1.5 hours. The filters were washed again and then developed using western blotting luminal reagent (SC-2048, Santa Cruz Biotechnology) and exposed to film in darkroom for visualization.

### ChIP assay

To crosslink chromatin, the cells were treated with 1% formaldehyde at room temperature for 10 min. The reaction was stopped by the addition of glycine to a final concentration of 125 mM. The cells were washed with PBS and then the cell pellet was resuspended in SDS lysis buffer (1% SDS, 10 mM EDTA and 50 mM Tris pH 8.1) containing protease inhibitor cocktail (Roche). For one experiment, 1 × 10^6 ^cells in 200 μl SDS lysis buffer were used for histone antibody and 1 × 10^7 ^cells in 200 μl SDS lysis buffer for transcription factor antibody. The crosslinked cell lysate was sonicated on ice until crosslinked chromatin was sheared to DNA fragment length between 0.2 and 1 kb. After centrifugation at 14 000 r.p.m. for 15 min, the 200 μl sonicated cell supernatant was diluted 10-fold by adding 1800 μl ChIP Dilution Buffer (0.01% SDS, 1.1% Triton X-100, 1.2 mM EDTA, 16.7 mM Tris-HCl pH 8.1, 167 mM NaCl, adding protease inhibitors as above). Meanwhile, another 20 μl diluted cell supernatant was considered as input to quantitate the amount of DNA in different samples but it needed reverse the histone-DNA crosslinks by heating at 65°C for 4 h. The 2 ml diluted cell supernatant was precleared by incubation with 80 μl of Salmon Sperm DNA/Protein A Agrose-50% Slurry (16–157, Upstate) with agitation at 4°C for 30 min. The precleared chromatin was incubated with 4 μg antibody with rotation at 4°C overnight. All the used antibodies were the rabbit polyclonal antibodies against H3 acetylation (06–599), H4 acetylation (06–866), H3-K4 dimethylation (07–030), H3-K79 dimethylation (07–366) (Upstate), and against NF-E2 (C-19, Santa Cruz Biotechnology). For negative control, a no-antibody immunoprecipitation was performed by incubation of the supernatant fraction with 60 μl of Salmon Sperm DNA/Protein A Agrose-50% Slurry (The results was not shown). Immunoprecipitation was recovered by incubation with 60 μl of Salmon Sperm DNA/Protein A Agrose-50% Slurry at 4°C with rotation for 1 h, followed by gentle centrifugation. The protein A agarose/antibody/histone complex was pelleted and washed with 1 ml of each of the wash buffers for 5–10 min including low salt buffer, high salt buffer, and LiCl buffer (Upstate). Finally, it was washed twice using 1× TE. Then the pelleted protein A agarose/antibody/histone complex was added 250 μl freshly prepared elution buffer (1%SDS, 0.1 M NaHCO_3_) and then incubated at room temperature with rotation for 15 min to elute the histone complex from antibody. The supernatant fraction (eluate) was transferred to another tube and elution was repeated. The ~500 μl eluate was obtained to reverse crosslinks by incubation at 65°C for 6 h after adding 20 μl 5 M NaCl. The 10 μl of 0.5 M EDTA, 20 μl of 1 M Tris-HCl, PH6.5 and 2 μl of 10 mg/ml Proteinase K (Roche) were added to the eluate and then it was incubated at 45°C for 4–5 h. The DNA was recovered by phenol/chloroform extraction and ethanol precipitation (adding 20 μg glycogen). The pellet was washed with 70% ethanol and dried in air. The dried pellet was diluted in 50 μl ddH_2_O for PCR analysis. The primers used for ChIP analysis are listed in table 1.

PCR products were resolved by agarose-gel electrophoresis and revealed by staining with ethidium bromide. For the comparative ChIP analysis, the linear range of amplification was determined for every sample by serial dilutions. An appropriate amount of DNA within the linear range was subsequently applied. All the comparative ChIP analyses were performed three repeats independently.

## Authors' contributions

LX conceived of the study, carried out experimental design, and participated in and molecular genetic study and drafted the manuscript. GLZ participated in the experimental design as well as carried out molecular genetic study and drafted the manuscript. XSW participated in cell synchronization and ChIP analysis, help to draft the manuscript. GHW participated in immunofluoresence study. WS participated in immunofluoresence study. DLH carried out cell culture. DPL conceived of the study, participated in coordination and helped to draft the manuscript. CCL coordinated and helped to draft the manuscript.

## Figures and Tables

**Table 1:** The primers and primer sequences used in ChIP assay.

Primer name	Primer sequences	Products
c-myc	5'-CTGTCCTGGCTCGCAGATTGTAAG-3' 5'-AGTGAGCGGACGGTTGGAAGAG-3'	231 bp
hsp70	5'-GTCGCAGCCAATCAACATAAGGATG-3' 5'-GAAAGAGGCAGGAAACAAGTGACGC-3'	229 bp
β-actin	5'-GGACCGGCAACGAAGGAGCT-3' 5'-GTCCACAAGGGCGGAGGCTAT-3'	360 bp
α-globin	5'-TTTGGTTTGAGGGACTTGCTTC-3' 5'-TCATTGGCTGGTTTGTGCTTG-3'	210 bp
βmaj	5'-ACCGAAGCCTGATTCCGTAGAGC-3' 5'-GATGTCTGTTTCTGGGGTTGTGAGTC-3'	269 bp
7SK	5'-ACAGTCGGCTACATAAGGTCACCAC-3' 5'-AGCTTCGGGAGGGACGCACA-3'	213 bp
nef-3	5'-TGATAAAGAACAGCGGACCCTACG-3' 5'-AGTTCCAGACCTCTTGTCACCCG-3'	228 bp
albumin	5'-CAGATGGCAAACATACGCAAGG-3' 5'-GCAGAGCCGGAGACGAAGAG-3'	246 bp
HS26	5'-CGAGCAGATAACTAAGCCAAGC-3' 5'-TTAAGGTAACAGGCACAGGGTC-3'	185 bp
HS21	5'-TGCCAGAATAACATCCGAGAACC-3' 5'-GAGAGTCACTGATAGGAATGCCACC-3'	171 bp
HS8	5'-CCTTATCTGTCCCGTCACTTGCTC-3' 5'-ATTAATCTCGTCCATTCAGTTTCACC-3'	250 bp
HS3	5'-TGTTTCCCTGATGAGGATTCAATGG-3' 5'-CCCACACATGGTCATCTATCTGAGC-3'	274 bp
HS2	5'-TTCCTACACATTAACGAGCCTCTGC-3' 5'-AACATCTGGCCACACACCCTAAGC-3'	279 bp
HS1	5'-GCCTTACATCCCTGGACAGATAAAC-3' 5'-GTGACATTGTGAGAAACTGAGACCC-3'	342 bp
